# Study on the Infection Mechanism of *Penicillium Digitatum* on Postharvest Citrus (*Citrus Reticulata* Blanco) Based on Transcriptomics

**DOI:** 10.3390/microorganisms7120672

**Published:** 2019-12-10

**Authors:** Qiya Yang, Xin Qian, Solairaj Dhanasekaran, Nana Adwoa Serwah Boateng, Xueli Yan, Huimin Zhu, Fangtao He, Hongyin Zhang

**Affiliations:** 1 School of Food and Biological Engineering, Jiangsu University, Zhenjiang 212013, China; 2 Institute of Life Sciences, Jiangsu University, Zhenjiang 212013, China

**Keywords:** *Penicillium digitatum*, citrus, infection mechanism, transcriptomics, pathogenic factors

## Abstract

*Penicillium digitatum* is one of the most important pathogens known widely to cause postharvest losses of citrus. It is significant to explore its infection mechanism to improve the control technology of postharvest diseases of citrus. This research aimed to study the changes in gene expression of *P. digitatum* at its early stages of citrus infection by transcriptomics sequencing and bioinformatics analysis in order to explore the molecular mechanism of its infection. The results showed that genes associated with pathogenic factors, such as cell wall degrading enzymes, ethylene, organic acids, and effectors, were significantly up-regulated. Concurrently, genes related to anti-oxidation and iron transport were equally up-regulated at varying degrees. From this study, we demonstrated a simple blueprint for the infection mechanism of *P. digitatum* in *Citrus reticulata* Blanco, which provided a new direction for subsequent pathological research and paves the way for developing new control strategies.

## 1. Introduction

Citrus fruits are a high energy hyperalimentation group of fruits widely planted and consumed worldwide, which have significant impacts on human health. Among them, tangerine (*Citrus reticulata* Blanco) is one of the most important commercial citrus fruits in China. Secondary metabolites of tangerine, including flavonoids, alkaloids, limonoids, coumarins, carotenoids, phenolic acids, and essential oils, are of great significance to human health due to their functions [1]. Postharvest decay is the most serious cause of postharvest loss of citrus in the world. Green mold and blue mold caused by *Penicillium digitatum* and *Penicillium italicum* are two main types of postharvest decay of citrus, usually causing 90% of the total loss [2]. Between them, *P. digitatum* is also known to produce a potential mycotoxin named citrinin, which has nephrotoxicity, embryotoxicity, teratogenicity, and carcinogenicity effects on humans and animals [3].

Postharvest handlings, like avoiding mechanical damage during harvest and transportation and maintaining good storage and transportation conditions, can effectively reduce postharvest decay of citrus [1]. Still, a lack of supporting facilities in the cultivation fields, like high cost refrigeration storage and optimum transportation system, makes the chemical fungicides approach as an efficient and cheap first port of call by fruit farmers. However, emergence of resistant fungal populations towards chemical fungicides such as thiabendazole, imazalil, and sodium o-phenylphenate [4] are coupled with harm to the environment and human health [5]. Hence, people are increasingly demanding for the development of safe and effective non-chemical control methods. However, the efficiency of the developed non-chemical techniques still cannot achieve the efficacy of chemical fungicides, and its action mechanism has not been elucidated [6]. Development of more targeted and efficient non-chemical-based alternatives require a thorough pathogenic understanding of the underlying mechanisms by which pathogens infect postharvest citrus fruits.

*P. digitatum* is a necrotrophic pathogen of citrus that can infect only through the wounds on the surfaces of fruits. The spores of *P. digitatum* can germinate and grow rapidly after coming into contact with the wound tissue, and completes its growth cycle within two days, resulting in citrus soft rot. After sequencing the mitochondrial genome of *P. digitatum*, Wang et al. knocked out a series of genes related to its growth and development, cell wall degrading enzyme (CWDE), and other genes to prove to their pathogenicity [7]. However, whether destroys the growth of *P. digitatum* or reduces the secretion of its pathogenic metabolites, it does not prevent the infection of *P. digitatum* completely in citrus. In recent years, with the extensive application of transcriptomics in plant pathology research, a wide range of research work was carried out to study postharvest diseases of fruits [8]. For example, 3206 differentially expressed genes (DEGs) were identified by transcriptome sequencing during the spore germination of *Penicillium expansum*, and the molecular process of the germination of *P. expansum* was deciphered by proteomic binding analysis [9]. Wang et al. analyzed the molecular changes of CWDEs, antioxidant stress, pH regulation, and effectors during initial infection by transcriptome sequencing of *P. expansum* [10]. The results showed that *P. expansum* could promote its infection on apples by reducing the pH of wounds, and secrete some antioxidants and effectors to resist the host’s defense response. However, *P. digitatum* is also reported as “acidic fungus,” which may have a similar mechanism to *P. expansum* in the infection process [11].

Transcriptomics can comprehensively and intuitively display the gene expression of pathogenic fungi in the process of infection, and thus can similarly delineate the molecular mechanism of *P. digitatum* infection in postharvest citrus. In our previous work, through microscopic observation, enzyme activity, and physical and chemical determination, the key time point (44 h post-inoculation) of *P. digitatum* infection on citrus was determined [12]. In this study, we performed high-throughput transcriptome sequencing of *P. digitatum* in the early stages of infection (0–44 h post-inoculation). The accuracy of the results was verified by qRT-PCR. We intended to study the transcriptional level changes of *P. digitatum* before infection and during postharvest citrus decay. The DEGs and their related metabolic pathways were analyzed by several bioinformatics tools. Overall, the study provided some molecular insights on the infection mechanism of *P. digitatum* on postharvest citrus.

## 2. Materials and Methods

### 2.1. Pathogen

The pathogen was isolated in our previous work and identified as *P. digitatum* [12]. The *P. digitatum* were cultured on potato dextrose agar (PDA) medium at 25 °C for 7 days, then spores were collected and the concentration was adjusted to 1 × 10^6^ spores/mL suspension with normal saline.

### 2.2. Fruit

Tangerine (*Citrus reticulata* Blanco) fruit with commercial maturity, similar color and sizes, and no mechanical damages on their surfaces were used in this research. The fruit were soaked in 0.1% (*w*/*v*) NaClO for 2 min, rinsed under running water, allowed to dry naturally, and used for further experiments.

### 2.3. Sample Preparation

Three wounds (3 mm wide by 5 mm deep) were created uniformly at the equator of the citrus fruit with a sterile puncher. Thirty microliters of the prepared spore suspension of *P. digitatum* was inoculated into each wound. The inoculated fruits were placed in baskets and wrapped with transparent polyethene to maintain 95% relative humidity and stored at 25 °C. The tissue samples (about 3 g) were collected from the wound after 44 h post inoculation (hpi). They were immediately pre-cooled with liquid nitrogen, and stored at −80 °C for subsequent analysis. *Penicillium digitatum* spores that were frozen immediately after culturing on PDA medium were prepared as a control. Two replicates were prepared for each sample.

### 2.4. RNA Extraction

The sample was ground into fine powder in liquid nitrogen, and then total RNA was extracted according to the method shown in the column fungus total RNA extraction kit (Sangon. Co., Shanghai, China). The purity and concentration of the RNA samples were determined using a One Drop OD-1000+ spectrophotometer (Wuyi science and Technology Co., Ltd., Nanjing, China). RNA integrity was then detected with the Bioanalyzer 2100 (Agilent Technologies Co., Santa Clara, CA, USA).

### 2.5. High Throughput Sequencing of Transcriptome

RNA samples were sequenced by the biotechnology corporation (Genepioneer Biotechnologies Co., Nanjing, China). After mRNA enrichment, double-strand cDNA synthesis, terminal repair and ligation, PCR enrichment, and library quality control, subsequent sequencing using Illumina HiSeq 2500 was done [13]. After data segmentation and quality control of the offline file after sequencing, the reference genome GCF_000315645.1_PdigPd1 (ftp://ftp.ncbi.nlm.nih.gov/genomes/all/GCF/000/315/645/GCF_000315645.1_PdigPd1_v1/GCF_000315645.1_PdigPd1_v1_genomic.fna.gz) was used to get the clean data.

### 2.6. Bioinformatic Analysis of RNA-Seq Data

The Benjamini–Hochberg correction method was used to correct the significant *p*-value obtained from the original hypothesis test to obtain the false discovery rate (FDR). The differentially expressed genes (DEGs) were obtained using |log2(Fold Change)| ≥ 1 and FDR < 0.05 as screening criteria. Gene databases such as gene ontology (GO) [14], cluster of original groups of proteins (COG) [15], Kyoto encyclopedia of genes and genes (KEGG) [16], SwissProt [17], and National Center for Biotechnology Information (NCBI) [18] were used to annotate the functions of DEGs.

### 2.7. Validation of RNA-Seq Data by qRT-PCR

Twenty DEGs were randomly selected from RNA-seq data, and qRT-PCR analysis and validation of these DEGs were performed on samples. The designed specific primers are shown in Table S1, with a reaction system: 10 μL TB Green; 0.4 μL 50 × Rox Reference Dye II; 2 μL Primer-F; 2 μL Primer-R; 2 μL cDNA; 3.6 μL ddH_2_O. Analysis was performed using QuantStudio 3 System (Thermo Fisher Scientific Inc., Waltham, MA, USA) (Step 1: 95 °C, 10 s; Step 2: 95 °C, 5 s; Tm −5 °C, 31 s; Step 2 cycle for 35 times). Among them, a β-tublin gene of *P. digitatum* was used as an internal reference, and the relative expression level of the sample gene was calculated using a 2^−ΔΔCT^ method. Each sample consisted of three parallels and the entire experiment was repeated three times.

## 3. Results

### 3.1. Overview of RNA-Seq Data

**Figure 1:**
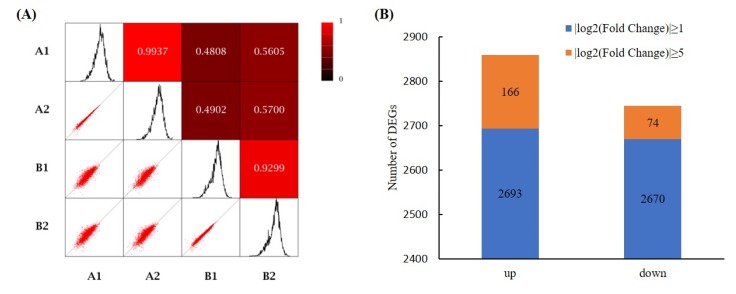
Overview of RNA-seq data. (**A**) Pearson’s correlation coefficient, A1 and A2 are two replicates of *P. digitatum* spores that were frozen immediately after culture on potato dextrose agar (PDA) medium, B1 and B2 are two replicates of *P. digitatum* spores that have been inoculated on citrus wounds for 44 h; (**B**) Statistics of differentially expressed genes.

The raw data of RNA-seq for 0 and 44 hpi samples were uploaded to NCBI’s Gene Expression Omnibus (GEO) database, which we obtained with the accession number GSE128979. The Pearson’s correlation coefficient (*r*) of the two biological replicates in the experimental group (44 hpi) reached 0.9299, and in the control group (0 hpi) was 0.9937, whereas between the experimental group and the control group was 0.5254 (Figure 1A).

As shown in Figure 1B, using |log2(Fold Change)| ≥ 1 and FDR < 0.05 as screening criteria, 5363 genes were identified as DEGs, among which 2693 were up-regulated and 2670 were down-regulated. By using |log2(Fold Change)| ≥ 5 as screening criteria, 240 genes were identified as DEGs, among which 166 were up-regulated and 74 were down-regulated.

### 3.2. Validation of RNA-Seq Data by qRT-PCR

**Figure 2:**
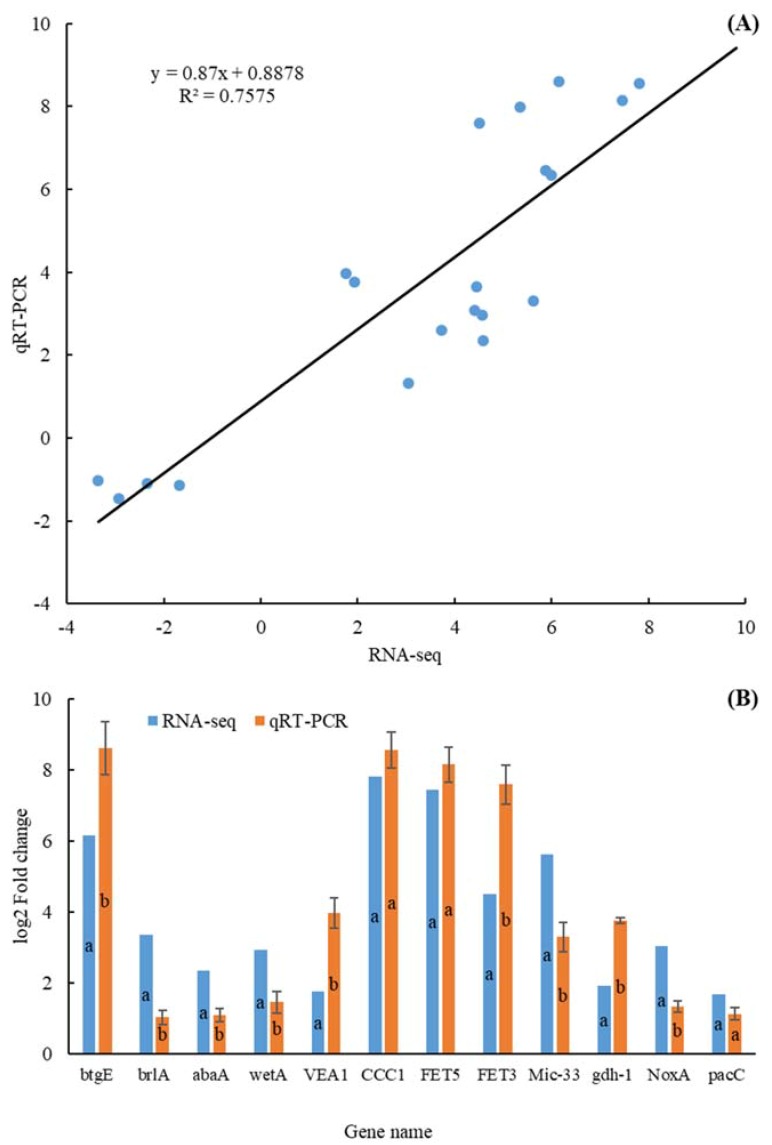
The comparison of gene expression values obtained by qRT-PCR and RNA-seq. qRT-PCR: The gene expression log2(Fold Change) value of 20 differentially expressed genes (DEGs) in qRT-PCR analysis; RNA-seq: The gene expression log2(Fold Change) value of DEGs in RNA-seq analysis; (**A**): linear relationship; (**B**): numerical comparison; Data in columns with the different letters are significantly different according to Duncan’s multiple range test at *p* < 0.05.

In order to verify the reliability of RNA-seq results, the relative expression of 20 randomly selected DEGs was determined by qRT-PCR and compared with RNA-seq data. The results showed that the up- and down-regulation of the verified genes were consistent, and the overall correlation coefficient R^2^ was 0.76 (Figure 2). The genes selected and the specific primers used are shown in Table S1.

### 3.3. Clustering and Functional Enrichment of DEGs

After annotating with multiple gene databases (Table S2), the annotation results were clustered and enriched.

**Figure 3:**
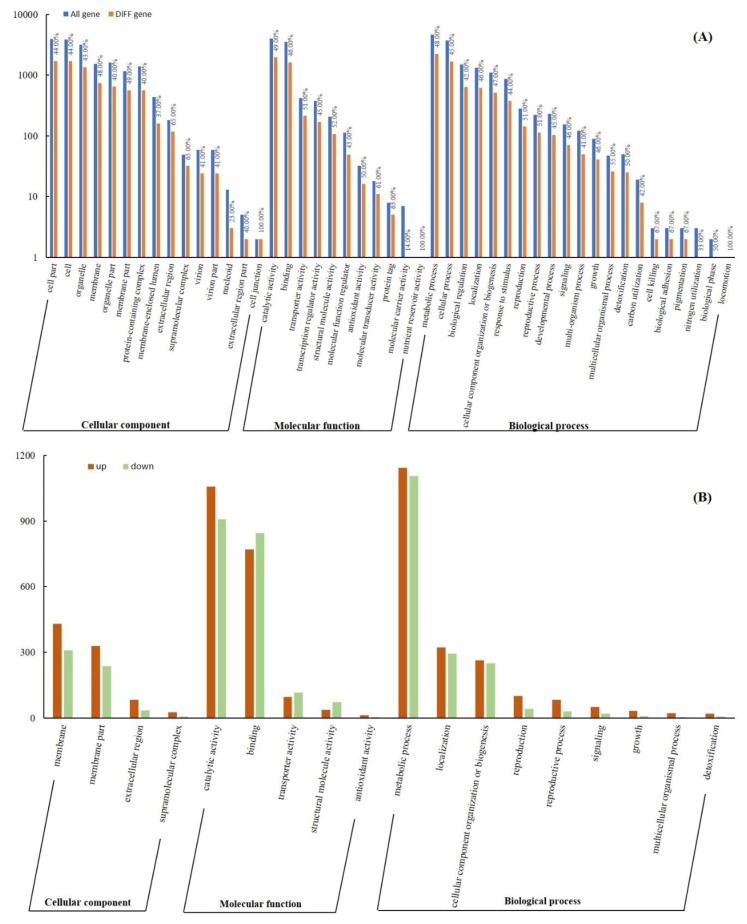

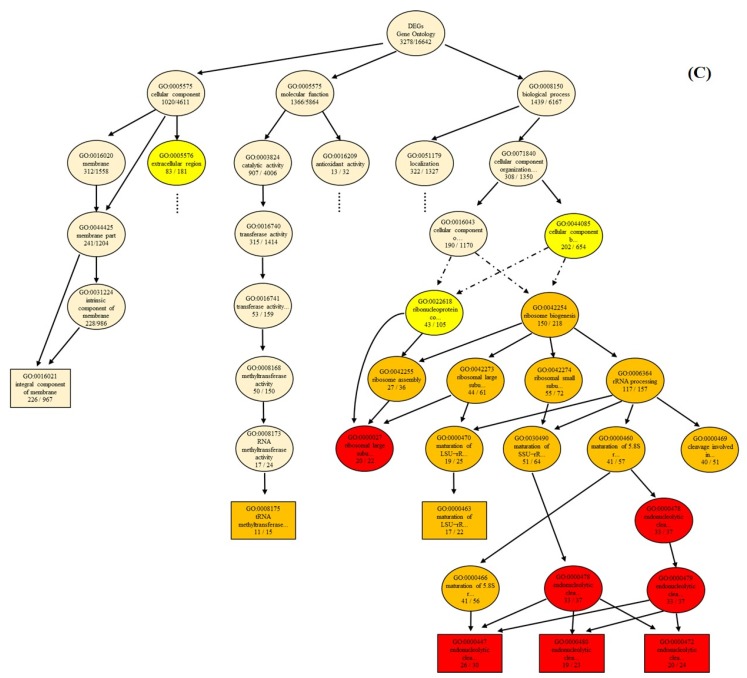
Gene ontology (Go) enrichment classification of DEGs. (**A**) Go classification of all genes and DEGs in RNA-seq results; (**B**) Analysis of key GO terms; (**C**) TopGO analysis of key GO term.

The secondary classification enrichment analysis of GO showed that DEGs were clustered into 15 terms of cellular components, 11 terms of molecular function, and 21 terms of biological processes (Figure 3A). Among them, there are 18 terms (13 up-regulations more than down-regulations) whose difference rates are higher than the basic difference rate (45.49% = ∑DEGs/∑All gene) and the number of DEGs is larger than 10 (Figure 3B). Further topGO level analysis was performed on several more function classes of concern. The results showed that the up-regulated expression rate of DEGs in endogenetic cleavage involved in rRNA processing (GO: 0000478) was more than 80% (Figure 3C).

After clustering the DEGs according to COG database (Figure S1), most genes were clustered to seven functional classes such as amino acid transport and metabolism (296), carbohydrate transport and metabolism (267), inorganic ion transport and metabolism (204), translation, ribosomal structure and biogenesis (164), replication, recombination, and repair (151), secondary metabolites biosynthesis, transport, and catabolism (144), and energy production and conversion (132).

**Figure 4:**
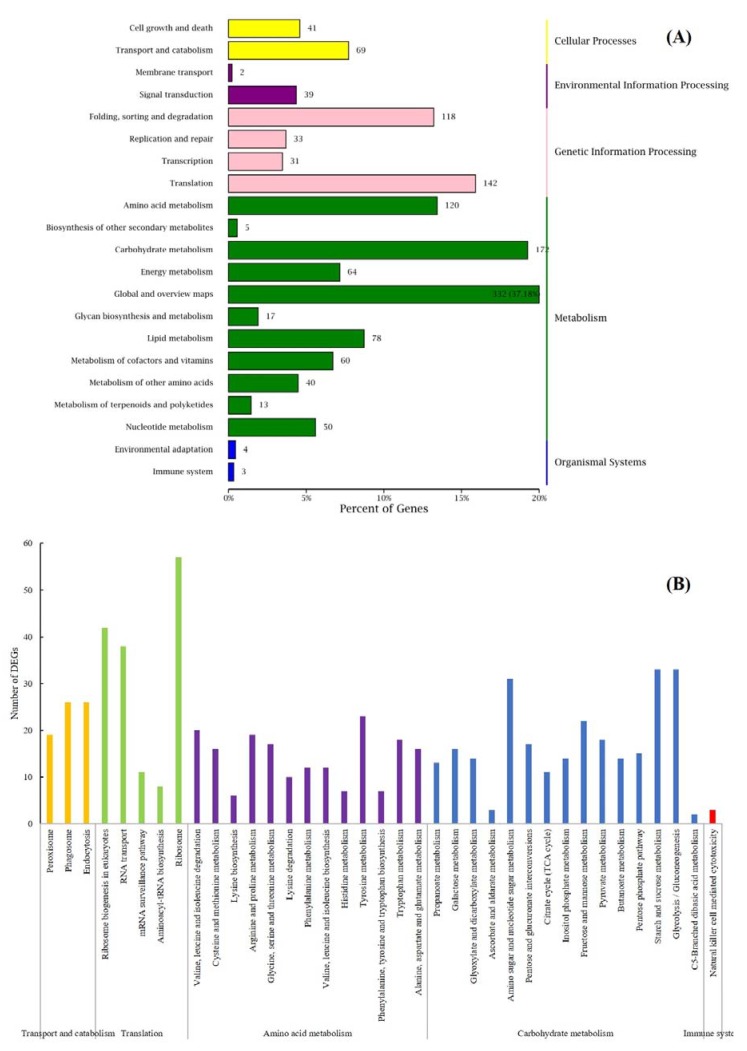
Kyoto encyclopedia of genes and genes (KEGG) enrichment classification of DEGs. (**A**) KEGG secondary classification of all DEGs; (**B**) KEGG third level classification of key KEGG pathway.

The cluster analysis of KEGG showed that environmental information processing, metadata, organizational systems, cellular processes, and genetic information processing were respectively clustered with 41, 481, 7, 110, and 316 DEGs (Figure 4A). Transport and catabolism, translation, amino acid metabolism, carbohydrate metabolism, and immune system were selected for the next level of enrichment classification (Figure 4B). From the results, we confirmed that 120 DEGs were clustered into 13 types of amino acid metabolism pathways which involved the degradation and synthesis of 18 different amino acids. In addition, 172 DEGs were clustered into 15 types of carbohydrate metabolism pathways, of which most DEGs were enriched in starch and sucrose metabolism and glycolysis/glycogenesis.

### 3.4. Analysis of Key DEGs of P. Digitatum in Infection Process

After annotation and enrichment analysis using multiple genomic databases, much concern was required to be given to some key DEGs. The key DEGs were mainly involved in spore growth, CWDEs, pH, iron transport, ethylene synthesis, anti-oxidative stress, and effectors.

The expression of genes related to spore germination, conidiophore development, and conidia formation are shown in Table S3. The expression of most of the genes involved in fungal development were decreased, with the exception of PDIP_21430 and PDIP_64730 which were related to oxidative stresses and were up-regulated to 3.39 and 3.10 times, respectively.

CWDEs are the most frequently reported pathogenic factors of plant pathogens. The large number of genes, associated with the synthesis of CWDEs, found in this study were already reported in our previous study [12]. In addition, the pH of the host environment is closely related to the synthesis and activity of CWDEs. Five DEGs, which included PDIP_86430 and PDIP_57180, related to the synthesis of organic acids were also found in the present study and are shown in Table S4.

Iron is essential for fungal growth and pathogenesis [5], and ethylene contributes to the growth of a variety of filamentous pathogenic fungi [19]. DEGs related to iron transport and ethylene-forming enzyme (EFE) are shown in Tables S5 and S6, respectively. In addition, 14 genes associated with prevention of oxidative stress are listed and counted in Table S7. Other effectors such as necrosis-inducing protein (PDIP_77830), killer toxin subunits (PDIP_06030), etc. are listed in Table S8.

## 4. Discussion

Green mold is the disease that causes the greatest loss of postharvest citrus [2]. At present, transcriptome analysis has been used in the pathological study of postharvest blue mold disease caused by *P. expansum* in apples, and some impressive results have been achieved [8,9,10]. However, transcriptome analysis hasn’t been used to determine the infection mechanism of *P. digitatum* in citrus fruits and thus the molecular mechanism behind the infection process is lacking. Therefore, this study sampled the *P. digitatum* infection in citrus at key time points as revealed in our previous study [12], and explored the key genes and pathways through high-throughput transcriptome sequencing, that reveal its infection mechanism at the molecular level.

*P. digitatum* spores can activate rapidly and start colonization after they come into contact with citrus wounds. After the activation of spores, further fungal development such as germination of spores, growth of germ tubes, formation of conidiophore stacks, and phialides and new conidia were completed within two days [7,12]. At this time, the citrus wounds would also rot. We have performed RNA-seq at 0 and 44 hpi of *P. digitatum*, and 20 DEGs selected randomly from the results and qRT-PCR was performed to validate them (Figure 2). The results proved that the differential expression observed in RNA-seq was consistent with qRT-PCR results. Concurrently, the Pearson’s correlation coefficient between the biologically repeated samples of RNA-seq was higher (r = 0.9299). This indicated that the results of RNA-seq was accurate and reliable, enabling subsequent analysis.

The growth process of *P. digitatum* is roughly broken down into spore germination, germ tube growth, conidiophore stalks differentiation, and phialides and new conidia formation [10]. The genes *brlA*, *abaA*, *wetA*, *vosA*, *VEA1*, and *VelB* have been shown to control the different steps of fungal growth and development, and the deletion of any gene will lead to growth defects and loss of pathogenicity of pathogens [7,20]. In the RNA-seq results of this study, *PdbrlA* (PDIP_05330), *PdabaA* (PDIP_20340), *PdwetA* (PDIP_44350), and *PdvosA* (PDIP_33270) were down-regulated in varying degrees (Table S3), which may be related to our sampling time and also indicates that *P. digitatum* completed its growth cycle in the wound. By contrast, the genes *PdVEA1* (PDIP_21430) and *PdVelB* (PDIP_64730) were up-regulated about 3.39 and 3.10 times, respectively, and were related not only to fungal development, but also to oxidative stress sensitivity. In the early stages of infection, the oxidative stress sensitivity should occur in *P. digitatum*, when the citrus fruit has a series of oxidative bursts in response to infection [21]. In order to prevent the damage caused by oxidative stress, up-regulation of these genes took place. In addition, more than 17 other DEGs involved in the prevention of oxidative stress were mentioned in Table S7, including glutathione S-transferase, which can mediate ROS resistance, and catalase, which can eliminate hydrogen peroxide.

In addition to the DEGs associated with the growth of *P. digitatum*, the focus was primarily on the virulence factors of *P. digitatum*. Cell wall degrading enzymes (CWDEs) are virulence factors which have been widely reported on phytopathogenic fungi [22]. CWDEs can help the pathogens to degrade plant cell walls, break through the host’s defense barrier, and obtain the nutrients needed for their growth. More than 20 up-regulated genes related CWDEs such as *PdPG1* (PDIP_64460) and *PdeglB* (PDIP_65210) were found in the joint analysis of extracellular region and catalytic activity (GO) (Figure 3), carbohydrate transport and metabolism (COG) (Figure S1), starch and sucrose metabolism, and pentose and glucuronate interconversions (KEGG) (Figure 4). The results provided more evidence for the importance of CWDEs in the pathogenesis of *P. digitatum* on postharvest citrus. The most important parameter to consider for the pathogen infection process is pH of the host environment, and lower pH of the environment that contributes to the synthesis of CWDEs and facilitates their function [23]. *P. digitatum* was reported as an “acidic fungus” [11], which secretes organic acids such as gluconic acid that reduces the pH of the surrounding environment and promotes the expression of genes related to the synthesis of pectinase in the process of infection [24].Through the combined analysis of multi-database clustering, DEGs involved in the synthesis of gluconic acid precursors, glucose oxidase and glucose-methanol-choline oxidoreductase, such as *PdpatE* (PDIP_86430) and *Pdgox* (PDIP_57180), were found to be up-regulated (Table S4).

Ethylene is a phytohormone, and some of pathogens produced ethylene during their process of infection to facilitate the pathogenesis on hosts [19,25]. There was some research that showed ethylene can act as a regulator of plant disease resistance [26]. In this study, RNA-seq results showed that at least 5 DEGs associated with the synthesis of ethylene-forming enzyme (EFE) were up-regulated (Table S1). Moreover, *P. digitatum* has been reported to produce ethylene during its growth both in vitro and in vivo [27]. Although ethylene plays different roles in different pathogen-plant systems, it was denoted as an infection marker for fruit diseases caused by some ethylene-producing pathogens [28]. Meanwhile, ethylene synthesis needed the participation of Fe^2+^. The 2-oxoglutarate-Fe (II) oxidase (*PDIP_83100*) in Table S6 was an EFE associated with Fe^2+^. In addition, iron (Fe) has been proved to be essential for fungal growth and pathogenesis. Other DEGs associated with iron transport and uptake were also significantly upregulated (Table S5). Limiting the iron metabolism greatly reduced the pathogenicity of pathogens to a large extent, and the phenomenon was confirmed in studies of the mechanism of action of some postharvest biocontrol agents [5].

In the process of infection, secretion of some pathogenic substances may help the pathogens to invade or even kill the host cells. In order to counteract the host’s pathogen-associated molecular patterns (PAMP) triggered immunity (PTI), the pathogen also secretes some effectors that interfere with PTI and inhibit the host’s defense response [29]. For example, *Cladosporium fulvum*, the pathogen of tomato leaf, inhibits the host’s basic defense by secreting the effector *Avr2* [30]. The gene *Cmu1* of *Ustulago maydis*, a pathogen of maize smut, can produce an effector that interferes with the synthesis of the plant defense signaling factor salicylic acid [31]. However, fungal effectors are often called “the sea of diversity”, that is, in the effectors of fungal pathogen showed diversified sequence and structure, and the effectors of different plant pathogens are specific to the pathogen [32]. Therefore, we have explored the effectors secreted by *P. digitatum* in the infection process of citrus as much as possible. In our results, two and three up-regulated DEGs were annotated as necrosis-inducing protein and containing LysM domain, respectively [Table S8]. Necrosis-inducing protein not only inhibits the host’s defense response, but even directly induces the death of host cells [33]. The effectors containing LysM domain can prevent the host from recognizing chitin [34,35,36], thereby inhibiting its defense response.

**Figure 5:**
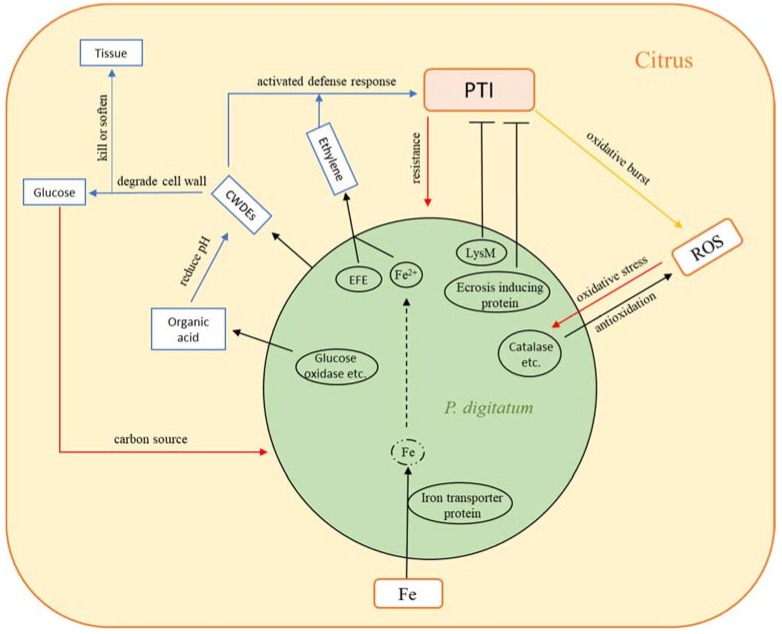
A simplified schematic diagram of the infection mechanism of *Penicillium digitatum* on citrus. After *P. digitatum* colonization in citrus wounds, it up-regulated the expression of the iron transporter protein for Fe absorption from the citrus wounds to meet its requirements for growth. Among them, the up-regulation of Fe^2+^ and ethylene-forming enzyme (EFE) in *P. digitatum* promoted ethylene synthesis. Ethylene is a growth and development promoter in *P. digitatum*. Again, genes that synthesize extracellular plant cell wall degrading enzymes and organic acid were upregulated by *P. digitatum* to promote degradation of plant cell walls into glucose and other carbon sources, providing nutrients necessary for growth. This process kills citrus cells and makes wound tissues rot. However, Cell wall degrading enzymes (CWDEs) and ethylene also activated PAMP-triggered immunity (PTI) of citrus to inhibit the infection of *P. digitatum*, which results in a series of oxidative bursts in citrus wounds. In order to resist oxidative stress, the expression of anti-oxidant related components such as catalase was up-regulated by *P. digitatum*. It is noteworthy that *P. digitatum* also secreted some effectors like LysM domain proteins and ecosis-inducing protein that inhibits the PTI of citrus.

To summarize, we have made a schematic diagram of the infection mechanism of *P. digitatum* on postharvest citrus (Figure 5). In addition, certain terpenoid secondary metabolites released from citrus peel can be served as signaling compounds in the host recognition procedure by *P. digitatum* [6]. In this study, the secondary metabolites’ biosynthesis-related genes of *P. digitatum* are up-regulated (Figure S1), which may have some connections with it, but more follow-up studies are needed to confirm.

## 5. Conclusions

In summary, this study unveiled the changes in gene expression of *P. digitatum* during the initial infection process on citrus by RNA-seq and subsequent bioinformatics analysis. DEGs related to the synthesis of organic acids, CWDEs and ethylene, iron transport, anti-oxidation, and effectors have been identified. The infection mechanism of *P. digitatum* in postharvest citrus was explored and a simple blueprint was drawn. In the follow-up experiments, we will further verify the function of some key genes identified in the present study to determine their role in the infection process of *P. digitatum*. The research will contribute to the development of new biological control methods for postharvest green mold on citrus according to the infection mechanism.

## Supplementary Materials

The following are available online at https://www.mdpi.com/2076-2607/7/12/672/s1.
