# Supplementary material for: Study on the Infection Mechanism of Penicillium Digitatum on Postharvest Citrus (Citrus Reticulata Blanco) Based on Transcriptomics
Source: Microorganisms. 2019 Dec 10;7(12):672. doi: 10.3390/microorganisms7120672 (PMC6956011; doi:10.3390/microorganisms7120672)
Supplement: Supplementary file 1 [file microorganisms-07-00672-s001.pdf]

microorganisms

Table S1. Gene specificity primers used in this study.

Gene Name	Primer	Primer Sequence (5' to 3')
pgaX	pgaX-F	TGGATGGTAACGGTCAGGTCTGG
	pgaX-R	GCGAGTAACGAAGGTTGAGAGGTC
eglB	eglB-F	CACTGTCACTGCCACTGCCAAG
	eglB-R	GGTCCAGTTGATGCCACCACAC
CELB	CELB-F	CGATGCCTCACTCACCAACACTG
	CELB-R	CAGCACGCAGGAGATGTCAAGAC
cbhA	cbhA-F	GTTGGCTCTCGTCTGTTCCTGATG
	cbhA-R	GCTCCATTGAGTCCGCAAGGC
galA	galA-F	TTGCGGCCAATCATCTCGATGTG
	galA-R	TGTTGCTGTAGCTGCTTGTTCCTC
manA	manA-F	CCTGGACTATGTTGTTGCCTCTGC
	manA-R	TAGGCGGCCATACCTCCATAATCC
xyn2	xyn2-F	CGATCACCACGAGCCAGCAAG
	xyn2-R	TCACCTGAAGCACCGTTGTTGTAG
abnA	abnA-F	CCGCCAGCGGAGCAGAATAC
	abnA-R	CAGGACCGTATACTGTGCCATGAC
btgE	btgE-F	GCTTCCTTCATCCAGTCCGCTTC
	btgE-R	GATGTCAACGGCACCGCAGAG
brlA	brlA-F	CGATGCCTCAGTACAACCAGATGG
	brlA-R	ATCGGACGAGACAAGCAAGGATTG
abaA	abaA-F	CCGTAACTTCTTCCGCTCCTTGTC
	abaA-R	GCCATACGCTTGCTCTCATCACC
wetA	wetA-F	GCCTTCTCAGTACGACGCAAGAC
	wetA-R	TGGCTGCCGTGCTGACATTG
VEA1	VEA1-F	CCACCGCCGATGATACGATTGC
	VEA1-R	GCCGATGTCAAGTTGGACGAGTAG
CCC1	CCC1-F	GGTGGTATTGCCGCTGGTTCTG
	CCC1-R	GTCATGCTTGTGGTCCTCGTGAG
FET5	FET5-F	GTCCACAAGCCAGCATCCAGATC
	FET5-R	TGCTGCATCGACGCTGTCAAC
FET3	FET3-F	GTGGATGTTGCCTCTGACCTTGTC
	FET3-R	CCGCACCGTTCCGTAGATTGTTC
Mic-33	Mic-33-F	ACGCAACCAAGACCGCTATTGAG
	Mic-33-R	GCCAGTCCGCCATTGAAGAAGG
gdh-1	gdh-1-F	AACGAGCACGACGAGATGGTTATG
	gdh-1-R	CGGAACTCAGAGCTGATGACAAGG
NoxA	NoxA-F	GGCTACGACGTGTCGAGTTCATC
	NoxA-R	CAGCGGTGTCAGCGTCAAGG

pacC	pacC-F	CGCACAGTCATACACCTCCAACC
	pacC-R	GGTCATACGCAACGCTCAGAGTAG
β-tublin	Tub-F	GATTGTCTTGAGAAATACTG
	Tub-R	TGTACCAGTGCAAGAAAGCC

Table S2. Number of differentially expressed genes annotated.

DEG Set	Total	Swiss-Prot	GO	KEGG	COG	KOG	Pfam	NR
A vs B	4582	3100	3278	1219	1891	2360	3061	4565

Table S3. The DEGs related to growth of *Penicillium digitatum* spores.

Gene ID	Gene Name	Function Classification	Description	Fold Change
PDIP_05330	*brlA*	growth	conidiophore stalks	–3.35
PDIP_20340	*abaA*	growth	phialides	–2.35
PDIP_44350	*wetA*	growth	conidiospore	–2.92
PDIP_21430	*VEA1*	growth; oxidative stress	growth; oxidative stress	1.76
PDIP_64730	*VelB*	growth; oxidative stress	growth; oxidative stress	1.63
PDIP_33270	*vosA*	growth	conidiospore	–1.58

Table S4. The up-regulated DEGs related to pH.

Gene ID	Gene Name	Function Classification	Description	Fold Change
PDIP_57180	*gox*	Organic acid	Glucose oxidase	2.53
PDIP_86430	*patE*	Precursors for organic acid	glucose-methanol-choline oxidoreductase	5.72
PDIP_54170	*xptC*	Precursors for organic acid	glucose-methanol-choline oxidoreductase	2.33
PDIP_23540	*patE*	Precursors for organic acid	glucose-methanol-choline oxidoreductase	2.08
PDIP_84520	*citC*	Precursors for organic acid	glucose-methanol-choline oxidoreductase	1.05

Table S5. The DEGs related to iron transport.

Gene ID	Gene Name	Function Classification	Description	Fold Change
PDIP_76510	*CCC1*	Iron storage	Transport protein	7.81
PDIP_65530	*FET5*	Iron uptake rate	Iron transport multicopper oxidase	7.45
PDIP_59000	*FET3*	Iron uptake rate	Iron transport multicopper oxidase	4.51
PDIP_44440	*NRPS12*	Synthesis of iron carrier	Non ribosome peptide synthetase	3.35
PDIP_39920	*NRPS11*	Synthesis of iron carrier	Non ribosome peptide synthetase	-1.03

Table S6. The up-regulated DEGs related to ethylene.

Gene ID	Gene Name	Function Classification	Description	Fold Change
MSTRG.6263	*AAT2*	Ethylene synthesis	Aspartate amino transferase	3.35
PDIP_36570	*Mic-33*	Precursors for ethylene	2-oxoglutarate carrier protein	5.62
PDIP_06610	*htyE*	Ethylene forming enzyme (EFE)	2-oxoglutarate	1.70
PDIP_83100	*encD*	Ethylene forming enzyme (EFE)	2-oxoglutarate-Fe(II)	1.93
PDIP_08660	*citB*	Ethylene forming enzyme (EFE)	2-oxoglutarate-Fe(II)	1.14
PDIP_32270	*gdh-1*	Precursors for ethylene	generate 2-oxoglutarate	1.93

Table S7. The up-regulated DEGs related to anti-oxidative stress.

Gene ID	Gene Name	Function Classification	Description	Fold Change
PDIP_07850	*tef3*	Glutathione S-transferase	Elongation factor 1-gamma	8.20
PDIP_64830	*hpm2*	Glutathione S-transferase	Transferase activity	5.11
PDIP_62390	*GTT1*	Glutathione S-transferase	Transferase activity	3.37
PDIP_06860	*ustS*	Glutathione S-transferase	Glutathione S-transferase-like protein	3.15
PDIP_12980	*FUS2*	Glutathione S-transferase	20-hydroxy-prefusarin hydrolase	2.99
PDIP_26410	*mtx1*	Glutathione S-transferase	Metaxin-1	2.08
PDIP_11910	*FUS3*	Glutathione S-transferase	Glutathione S-transferase-like protein	1.43
PDIP_77260	*gedE*	Glutathione S-transferase	Glutathione S-transferase-like protein	1.34
PDIP_74290	*tpcF*	Glutathione S-transferase	Glutathione transferase activity	1.32
PDIP_74730	*tpcF*	Glutathione S-transferase	Glutathione S-transferase-like protein	1.32
PDIP_80930	*gto2*	Glutathione S-transferase	Glutathione S-transferase omega-like	1.10
PDIP_86820	*unknow*	Catalase	oxidation-reduction process	3.15
PDIP_67580	*catB*	Catalase	Inorganic ion transport and metabolism	2.76
PDIP_05940	*PXP9*	Catalase	Peroxisomal catalase	1.63

Table S8. The up-regulated DEGs related to effectors.

Gene ID	Gene Name	Function Classification	Description	Fold Change
PDIP_77830	unknow	Necrosis inducing protein	Necrosis inducing protein	7.23
MSTRG.1774	unknow	Necrosis inducing protein	Necrosis inducing protein	6.25
PDIP_06030	unknow	LysM domain	Killer toxin subunits alpha/beta	4.55
PDIP_59730	unknow	LysM domain	Killer toxin subunits alpha/beta	1.11
MSTRG.8292	*chiA1*	Chitinase	Chitinase activity	6.00
PDIP_36560	*chiA*	Chitinase	Chitinase activity	5.62
PDIP_53900	*ctcA*	Chitinase	Chitinase activity	5.06

MSTRG.3881	*DAN4*	Chitinase	Chitinase activity	4.72
PDIP_79160	*chiA*	Chitinase	Chitinase activity	4.49
PDIP_84020	*chiA*	Chitinase	Chitinase activity	3.17
PDIP_85490	*chiA*	Chitinase	Chitinase activity	2.27
PDIP_16610	*chiA1*	Chitinase	Metal ion binding	2.06
PDIP_06200	*chiA*	Chitinase	Chitinase activity	1.70
PDIP_09650	*CHI1*	Chitinase	Chitinase activity	1.63
PDIP_83360	*chiB*	Chitinase	Chitinase activity	1.55
PDIP_53760	*CHI1*	Chitinase	Chitinase activity	1.39
PDIP_82800	*chiA1*	Chitinase	Chitinase activity	1.05

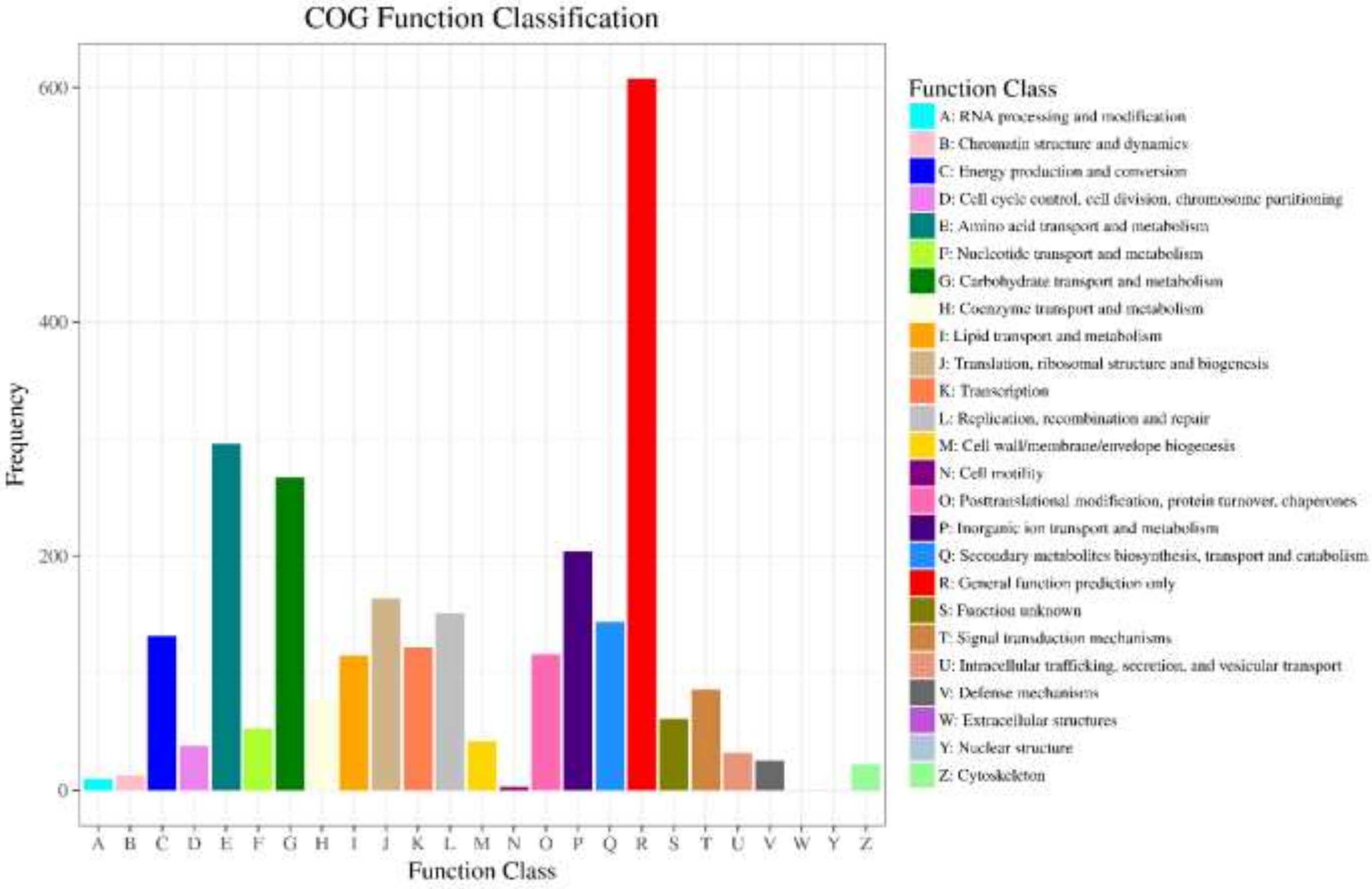

Figure S1. COG function classification of DEGs.
